# Association of BDNF and MMP-9 single-nucleotide polymorphisms with the clinical phenotype of schizophrenia

**DOI:** 10.3389/fpsyt.2022.941973

**Published:** 2022-10-17

**Authors:** Lihong Pan, Zhonghai Cao, Lianghu Chen, Min Qian, Yuzhong Yan

**Affiliations:** ^1^Pudong Nanhui Mental Health Center, Shanghai, China; ^2^People's Hospital of Datong County, Datong, China; ^3^Department of Research, Shanghai University of Medicine and Health Sciences Affiliated Zhoupu Hospital, Shanghai, China

**Keywords:** schizophrenia, BDNF, MMP-9, single-nucleotide polymorphisms, clinical phenotype

## Abstract

**Objective:**

Schizophrenia is a highly polygenic psychiatric disorder; however, the complex genetic architecture underlying the pathogenesis remains elusive. Brain-derived neurotrophic factor (BDNF), a neurotrophin, and matrix metalloproteinase 9 (MMP-9), a gelatinase B, are the promising candidate genes for schizophrenia. To shed new light on the relationship between the single-nucleotide polymorphisms (SNPs) of BDNF and MMP-9 and the clinical variability of schizophrenia phenotype, this study aims to evaluate the relationship, and provide more definitive evidence for the relationship with various clinical features of schizophrenia.

**Methods:**

A case-control association study was performed, and one hundred and five subjects of Chinese Han population were enrolled, including 55 schizophrenia patients (SP) and 50 healthy controls (HC). The BDNF rs6265 196 G > A and MMP-9 rs3918242 −1562C > T SNPs were genotyped using PCR-RFLP assay. The Positive and Negative Syndrome Scale (PANSS) was used to assess the clinical symptoms of patients with schizophrenia.

**Results:**

Compared with HC, the frequency of SP carrying BDNF rs6265 GG/GA genotype was significantly higher than HC, and the frequency of SP carrying BDNF rs6265 AA genotype was significantly lower than HC (*p* < 0.01). With regards to MMP-9 rs3918242 −1562C > T SNP, no significant difference was observed between the control and SP. BDNF GG genotype showed significantly higher PANSS and positive symptoms score than GA and AA genotypes (*P* < 0.01). MMP-9 CC genotype showed significantly higher PANSS and general score than CT and TT genotypes (*P* < 0.05).

**Conclusion:**

BDNF rs6265 196 G > A and MMP-9 rs3918242–1562C > T SNPs are related to the clinical features of schizophrenia and could be a useful biomarker for the changes, remission or deterioration of clinical status of schizophrenia.

## Introduction

Schizophrenia (SZ) is a highly polygenic psychiatric disorder with complex genetic architecture which affects about 1% of the general population worldwide, and it stimulates extensive research on the pathophysiology of this psychiatric disorder. A large amount of evidence suggests that insufficient neurotrophic supply of cortical neurons may be a potential factor in the pathophysiology of SZ, because normal brain development, maturation and function require adequate neurotrophic support (1, 2).

Brain derived neurotrophic factor (BDNF) is a member of the neurotrophic factor family. It is a key regulator of synaptic plasticity, which affects various brain functions, including cognition (3). Mature BDNF is initially synthesized as a precursor protein preproBDNF. After signal peptide cleavage, proBDNF is transformed into mature BDNF by extracellular protease, such as matrix metalloproteinase-9 (MMP-9) (4). Unbalanced expression of brain-derived neurotrophic factor (BDNF) and matrix metalloproteinase-9 (MMP-9) may affect neurogenesis, synaptic plasticity, learning and memory ability (4). Therefore, they can be regarded as diagnostic biomarkers. The report also showed that during the pathological process of the study, BDNF parameters decreased and MMP-9 expression increased and a negative correlation between them was noted (5). Pooled evidence suggested that genetic determinants could be related to SZ (6, 7). Converging lines of proof suggested that BDNF 196G > A SNPs and MMP-9 −1562C > T may be associated with SZ (6, 8–10). However, findings of earlier studies on the relationship of these SNPs with SZ are contradicting and it has not been definitively figured out which of these SNPs is associated with SZ.

In order to clarify the relationship between BDNF and MMP-9 single nucleotide polymorphisms and clinical variability of SZ phenotype, this study was aimed to evaluate the relationship between them and provide more conclusive evidence for their correlation with various clinical features of SZ.

## Materials and methods

### Subjects

This study group (*n* = 105) recruited 55 schizophrenia patients (SP) and 50 healthy controls (HC) from November 2018 to July 2021. All subjects enrolled were unrelated Han Chinese people. The diagnosis of schizophrenia was assigned by two independent psychiatrists based on the ICD-10 criteria. The group consisted of inpatients and outpatients. At the time of examination all patients were stabilized (no acute psychosis). The healthy control (HC) were recruited from the local health examination center. In addition, age-, gender- and smoking status matched healthy subjects who were ruled out the presence of a current/past psychiatric disorder, were recruited.

Exclusion criteria for patients were age <18 years, serious medical or surgical illness, previous episode of psychosis due to substance abuse, and psychotic symptomatology within a clearly diagnosed affective or borderline personality disorder. For healthy controls, exclusion criteria were age <18 years, current or past psychiatric disorder, family history of any psychiatric disorder, head trauma, neurological illness, serious medical or surgical illness, or substance abuse. Detailed medical information, such as demographics, sociodemographic characteristics, and medical conditions were obtained from all subjects. This study was approved by the Ethics Committee of Pudong Nanhui Mental Health Center, Shanghai, and either patients or their guardians signed informed consents.

### Clinical evaluation

Diagnosis and review of psychiatric case records were independently examined and assessed by at least two trained psychiatrists. Symptoms of schizophrenia were evaluated using the Positive and Negative Syndrome Scale (PANSS). The measurement of each patient's psychopathology was repeated twice to take the average value by two trained psychiatrists who were blind to the clinical status.

### Genotyping

Venous blood was collected from all subjects. Serum was separated and stored at −80°C until further analysis. Genomic DNA was extracted using QIAamp DNA Blood Kit (Qiagen, USA). The genotypes were determined using PCR-RFLP assay. A set of primers was designed from previously published articles (11, 12). Genotyping of the SNPs was analyzed by the Shanghai Major Biotechnology Co. Ltd. (www.majorbio.com).

### Statistical analyses

Statistical analyses were carried out using GraphPad Prism 6 software. Differences in clinical characteristics between patients and controls were analyzed using the Pearson's chi-square for categorical variables. Data were presented as mean ± standard deviation (SD) for continuous variables if normally distributed and compared by independent samples Student's *t*-test. Data was analyzed using one-way analysis of variance (ANOVA) with *post-hoc* comparisons. Fisher's exact test was used to analyze the significance of a difference between the proportions. A *P*-value of <0.05 was considered to be statistically significant.

## Results

### Demographic and clinical features

Demographic and clinical features for the subjects are shown in Table 1. There were no statistically significant differences regarding age (*P* = 0.33), gender (*P* = 0.78), smoking ratio (*P* = 0.74) and BMI (*P* = 0.64) between SZ patients and HC. The comparisons revealed less education for SZ patients than in the HC (*P* = 0.02). The antipsychotic treatment of the SZ patients are also listed in the table.

**Table 1:** Demographic and clinical characteristics of SZ patients and healthy controls.

	**SZ (*N* = 55)**	**HC (*N* = 50)**	**t/χ^2^**	***p*-value**
Age	33.22 ± 9.58	35.11 ± 10.13	0.98	0.33
Gender (M/F)	29/26	25/25	0.08	0.78
Education (years)	5.32 ± 2.99	6.73 ± 3.26	2.31	0.02
Smoking ratio (%)	30.90	28.00	0.11	0.74
BMI (kg/m2)	21.16 ± 3.12	20.89 ± 2.76	0.47	0.64
Age of onset (years)	27.12 ± 10.18			
Duration of illness (years)	9.19 ± 6.41			
**Antipsychotic treatment**				
Olanzapine (*N*)	33			
Risperidone (*N*)	18			
Aripiprazole (*N*)	3			
Chlorpromazine equivalents (mg/day)	503.55 ± 132.11			

### Association between BDNF and MMP-9 gene SNPs and SZ patients

The allele frequencies and genotype distributions of BDNF and MMP-9 gene SNPs are presented in Table 2. Concerning the BDNF rs6265 196 G > A SNP, the distribution of genotypes GG, GA and AA, were 32.73, 43.64, and 23.64% in SZ, respectively, whereas they were 10.00, 28.00, and 62.00% in HC, respectively. A statistically significant difference was indicated (*P* < 0.01) with the patients carrying a significantly higher frequency of the GG/GA genotypes and a lower frequency of the AA genotype in comparison to HC.

**Table 2:** Allelic distribution of BDNF and MMP-9 in SZ group and HC group.

	**Allele frequency (%)**		**Genotype distribution** ***N*** **(%)**			**HWE-*p***
**BDNF rs6265 196 G > A**	**G**	**A**	**GG**	**GA**	**AA**	
SZ	60 (54.55)	50 (45.45)	18 (32.73)	24 (43.64)	13 (23.64)	0.37
HC	28 (28.00)	72 (72.00)	5 (10.00)	14 (28.00)	31 (62.00)	0.1
	*x^2^* = 16.16, *p* < 0.01		*x^2^* = 17.14, *p* < 0.01			
**MMP-9 rs3918242-1562 C** > **T**	**C**	**T**	**CC**	**CT**	**TT**	
SZ	81 (73.64)	29 (26.36)	32 (58.18)	17 (30.91)	6 (10.91)	0.13
HC	70 (70.00)	30 (30.00)	27 (54.00)	16 (32.00)	7 (14.00)	0.09
	*x^2^* = 0.34, *p =* 0.56		*x^2^* = 0.29, *p =* 0.86			

HWE-p, the p-value of Hardy-Weinberg Equilibrium.

With regards to MMP-9 rs3918242 −1562C > T SNP, the distribution of genotype CC, CT and TT, was 58.18, 30.91, and 10.91% in SZ, respectively, whereas they were 54.00, 32.00, and 14.00% in HC, respectively. It was suggested that there was no significance between the controls and SZ patients.

### Relationship between BDNF and MMP-9 genotypes and clinical features of SZ patients

As shown in Table 3, there was no significant difference between the BDNF and MMP-9 genotypes with regards to age of onset and duration of illness. Concerning the genotypes of BDNF, the results indicated that GG genotype showed significantly higher PANSS (*P* < 0.01) and positive symptoms score (*P* < 0.01) than GA and AA genotypes, and in comparison with GA genotype, AA genotype also showed significant lower scores. On the other hand, the negative symptoms score (*P* = 0.21) and general score (*P* = 0.53) did not differ significantly among the three genotypes. As for the genotypes of MMP-9, the results indicated that CC genotype showed significantly higher PANSS (*P* = 0.03) and general score (*P* = 0.02) than CT and TT genotypes, but not in either positive (*P* = 0.52) or negative symptoms scores (*P* = 0.49).

**Table 3:** Relationship between BDNF and MMP-9 genotypes and clinical features of SZ patients.

	**Age of onset**		**Duration of illness**		**PANSS**		**Positive symptoms score**		**Negative symptoms score**		**General score**	
	**(years)**		**(years)**									
**BDNF rs6265 196 G** **>** **A**												
GG (*N* = 18)	25.11 ± 8.67	*F* = 0.31	10.23 ± 5.17	F = 0.16	75.26 ± 4.11	*F* = 8.06	23.36 ± 4.53	*F* = 4.23	25.87 ± 6.44	*F* = 1.63	32.55 ± 6.81	*F* = 0.64
GA (*N* = 24)	26.22 ± 8.13	*p =* 0.73	9.51 ± 4.29	*p =* 0.86	72.02 ± 4.38*	*p* < 0.01	20.54 ± 4.11_*_	*p <0.01*	23.10 ± 5.13	*p = 0.21*	31.01 ± 5.88	*P* = 0.53
AA (*N* = 13)	28.92 ± 7.76		8.51 ± 4.09		69.22 ± 3.89^***, #^		17.23 ± 5.08^**, #^		22.82 ± 4.92		30.11 ± 5.79	
**MMP-9 rs3918242−1562 C** **>** **T**												
CC (*N* = 32)	26.56 ± 7.45	*F* = 0.83	9.77 ± 4.42	*F* = 0.54	75.10 ± 5.36	*F* = 3.70	22.08 ± 6.03	*F* = 0.67	26.08 ± 7.24	*F* = 0.72	33.17 ± 3.84	*F* = 4.35
CT (*N* = 17)	27.67 ± 8.86	*p =* 0.44	9.23 ± 4.03	*p =* 0.59	72.01 ± 4.55*	*p =* 0.03	21.55 ± 5.09	*p = 0.52*	24.22 ± 6.31	*p* = 0.49	30.30 ± 4.01*	*p* = 0.02
TT (*N* = 6)	29.11 ± 6.89		8.92 ± 3.56		69.51 ± 4.89*		19.16 ± 5.28		23.13 ± 5.22		29.53 ± 3.32*	

*p < 0.05 GA vs. GG; **p < 0.01; ***p < 0.001 and ^#^*p* < 0.05 AA vs. GA; ^*$*^*p* < 0.05 CT vs. CC; ^&^*p* < 0.05 TT vs. CT.

## Discussion

Notwithstanding numerous groups endeavoring to examine the association between the BDNF rs6265 196 G > A and MMP-9 rs3918242 1562C > T SNPs and susceptibility to SZ, conflicting results remain. The purpose of this study was to determine whether BDNF and MMP-9 SNPs could be associated with the clinical variability of SZ phenotype. The first major finding of this study is that BDNF rs6265 SNPs, but not MMP-9 rs3918242 SNPs, were associated with SZ. It was suggested that allele (A) of rs6265 might confer a protection for SZ and correlate negatively with SZ. The second is that the BDNF and MMP-9 genotypes were related with the clinical features of SZ patients. The results demonstrated different significant scores among the genotypes, but were not related to age of onset or duration of illness.

Accumulating evidence proposed that many recently identified genes are potential risk factors in schizophrenia. BDNF and MMP-9 gene SNPs are the most intensively studied genes among them. Animal studies have shown that the homozygous BDNF mutant mice exhibit distinct behavioral phenotypes (13). They showed striking behavioral phenotypes of spinning, head bobbing and hindlimb extension, with deficiencies in coordination of movements and balance. Fu et al. (14) also demonstrated that a positive association was found between rs6265 and SZ. A meta-analysis conclusion is consistent with the results that the risk of SZ in individuals with the GG genotype was 19% higher than that in individuals with GA genotype (15). Regarding MMP-9 gene, a previous study (8) reported the prevalence of C/C genotype and C allele in SZ patients was significantly increased compared with the control group, which provided evidence for the significance of MMP-9 gene in the pathogenesis of SZ, and convincing indirect evidence showed that MMP-9 may be related to the pathological synaptic plasticity of SZ. To further clarify the role of BDNF and MMP-9 genes as risk alleles or regulators of schizophrenia, we carried out this case-control association study between BDNF rs6265 and MMP-9 rs3918242 SNPs. We reported a significant difference in the distribution of BDNF rs6265 SNP between SZ patients and HC patients, suggesting that BDNF SNP could be associated with SZ. Moreover, the hypothesis proposed that allele A of BDNF rs6265 confer a protection for schizophrenia. On the contrary, we reported that the distribution of the MMP-9 −1562C > T genotypes did not differ significantly between SZ patients and controls, suggesting that the MMP-9 −1562C > T SNP may not be associated with SZ. Furthermore, contrary to our findings, Han et al. (16) reported that SZ patients were more likely to be the T-allele carriers. Interestingly, the promoter −1562C > T SNP was shown to exert a functional effect on gene transcription, with the T allele showing a higher transcriptional activity than the C allele (17). Several studies have fixated on the association between MMP-9 genetic variants and pathogenesis of SZ. Especially, the role of the promoter −1562C > T SNP has been broadly studied, with strikingly contrasting results (6, 8, 16). More studies are also needed in order to evaluate the possible biomarker for changes in the clinical course of schizophrenia and response to antipsychotic treatment.

Despite the fact that numerous studies have been carried out on the assessment of the roles of BDNF and MMP-9 genotypes in association with clinical presentation, the results have become more contentious (18, 19). In this study, it was shown that the genotypes could play a critical role in SZ phenotype and contribute to the clinical expression. We found that significantly different PANSS and positive symptoms score were demonstrated among the genotypes of BDNF between SZ patients and HC. In line with our observations, Neves-Pereira et al. (20) also found an association between the BDNF rs6265 SNPs and the clinical presentation of schizophrenia on a Scottish population. It has been suggested that BDNF rs6265 genotype in hippocampus and inferior marginal medial prefrontal cortex may affect NMDA receptor-mediated neurotransmission and plasticity, which is related to the generation of positive or negative symptoms of schizophrenia (21). In addition, more and more evidence shows that BDNF rs6265 changes the clinical manifestation and genetic risk structure of schizophrenia, which may be achieved by affecting the clinical manifestation and treatment response (22). It has been suggested that more severe / chronic schizophrenia may be associated with allele (G), while less severe schizophrenia may be associated with allele (A) (10, 23, 24). Our data is consistent with the results presented above. Due to the lack of strong evidence and small sample size, studies describing the correlation between BDNF rs6265 and schizophrenia are still contradictory.

On the basis of Chinese Han population in this report, the assessment of the clinical significance of MMP-9 rs3918242 genotypes was also studied in the pathogenesis of SZ. Based on the data of this study, and in line with previous findings (7), MMP-9 1562 C/C genotype was considered as an independent predictor of the severity and was assessed by the positive and negative syndrome scale (PANSS), affirming association between MMP-9 genotypes and schizophrenia. Our results further support the hypothesis that MMP-9 could also play a critical role in the pathogenesis of SZ. Although in this study, we did not analyze the direct correlations between BDNF and MMP-9 genotypes, we assumed that there might be a compensated interaction between them as was previously reported (4). Taken together, our results suggested that the BDNF rs6265 196 G > A and MMP-9 rs3918242 −1562C > T SNPs could play a key role in the phenotype of SZ and contribute to the clinical presentation of SZ by altering a range of clinical features, including symptomatology.

The BDNF rs6265 196 G > A and MMP-9 rs3918242 −1562C > T SNPs are the most intensively studied genetic modifications in the psychopathology of SZ. However, it was clearly demonstrated in this report that BDNF rs6265 196 G > A and MMP-9 rs3918242 −1562C > T SNPs were related to the clinical phenotypes of SZ and could be useful biomarkers predicting the phenotypic expression and prognosis of SZ patients. It still remains contradictory in the absence of strong evidence, besides ethnic heterogeneity, sample size etc., which still need further investigation. In the absence of strong evidence, this is still contradictory. Our observation needs a confirmation through a large population study as the small sample size used for this study was a major limitation.

## Conclusion

The analysis of SNPs in the present study shows that BDNF rs6265 196 G > A SNP could be a biomarker for the differentiation of SZ patients and could be involved in the pathogenesis of SZ. Differences in genotype distribution of BDNF and MMP-9 might be responsible for the phenotypes of SZ which provide evidence on the pathophysiology of SZ, and it could eventually lead to better therapy.

## Data availability statement

The original contributions presented in the study are included in the article/supplementary material, further inquiries can be directed to the corresponding author.

## Ethics statement

The studies involving human participants were reviewed and approved by the Ethics Committee of Pudong Nanhui Mental Health Center, Shanghai. The patients/participants provided their written informed consent to participate in this study.

## Author contributions

YY: study conception, design, conceptualization, and drafting of the manuscript. LP and LC: patients recruitment and clinical monitoring. YY and ZC: conducting the biochemical and genotyping investigations. LC and ZC: acquisition of data. MQ and ZC: analysis and interpretation of data. LP, ZC, and YY: critical revision of the manuscript. All authors have approved the final version of the submitted manuscript.

## Funding

This study was supported by grants from Medical Discipline Construction Project of Pudong Health Committee of Shanghai (Grant No. PWYgts2021-05), the Research Grant for Science and Technology Commission of Pudong, Shanghai (Grant No. PKJ2019-Y61), and Shanghai University of Medicine and Health Science (Grant No. SFP-22-21-17-024). The funder had no role in study design, data collection and analysis, decision to publish, or preparation of the manuscript.

## Conflict of interest

The authors declare that the research was conducted in the absence of any commercial or financial relationships that could be construed as a potential conflict of interest.

## Publisher's note

All claims expressed in this article are solely those of the authors and do not necessarily represent those of their affiliated organizations, or those of the publisher, the editors and the reviewers. Any product that may be evaluated in this article, or claim that may be made by its manufacturer, is not guaranteed or endorsed by the publisher.
